# Obstinate Cases of Urethral Stricture

**Published:** 1844-06-29

**Authors:** 


OBSTINATE CASES OF URETHRAL STRICTURE.

In these cases Mr. Syme deprecated an attempt to effect dilatation by bougies, as no less dangerous than useless. He recommends division of the stricture, either by subcutaneous puncture, when it is seated in the pendulous part of the canal, or by a free incision upon a grooved director, when it lies behind the scrotum, as having proved completely successful in cases that had resisted every form of dilatation.

